# Genetic Linkage Mapping of Economically Important Traits in Cultivated Tetraploid Potato (*Solanum tuberosum* L.)

**DOI:** 10.1534/g3.115.019646

**Published:** 2015-09-14

**Authors:** Alicia N. Massa, Norma C. Manrique-Carpintero, Joseph J. Coombs, Daniel G. Zarka, Anne E. Boone, William W. Kirk, Christine A. Hackett, Glenn J. Bryan, David S. Douches

**Affiliations:** * Department of Plant, Soil and Microbial Sciences, Michigan State University, East Lansing, Michigan 48824; † Biomathematics and Statistics Scotland, Invergowrie, Dundee, DD2 5DA, United Kingdom; ‡ The James Hutton Institute, Invergowrie, Dundee, DD2 5DA, United Kingdom

**Keywords:** genetic linkage map, autotetraploid, potato, QTL, single-nucleotide polymorphism

## Abstract

The objective of this study was to construct a single nucleotide polymorphism (SNP)-based genetic map at the cultivated tetraploid level to locate quantitative trait loci (QTL) contributing to economically important traits in potato (*Solanum tuberosum* L.). The 156 F_1_ progeny and parents of a cross (MSL603) between “Jacqueline Lee” and “MSG227-2” were genotyped using the Infinium 8303 Potato Array. Furthermore, the progeny and parents were evaluated for foliar late blight reaction to isolates of the US-8 genotype of *Phytophthora infestans* (Mont.) de Bary and vine maturity. Linkage analyses and QTL mapping were performed using a novel approach that incorporates allele dosage information. The resulting genetic maps contained 1972 SNP markers with an average density of 1.36 marker per cM. QTL mapping identified the major source of late blight resistance in “Jacqueline Lee.” The best SNP marker mapped ∼0.54 Mb from a resistance hotspot on the long arm of chromosome 9. For vine maturity, the major-effect QTL was located on chromosome 5 with allelic effects from both parents. A candidate SNP marker for this trait mapped ∼0.25 Mb from the *StCDF1* gene, which is a candidate gene for the maturity trait. The identification of markers for *P. infestans* resistance will enable the introgression of multiple sources of resistance through marker-assisted selection. Moreover, the discovery of a QTL for late blight resistance not linked to the QTL for vine maturity provides the opportunity to use marker-assisted selection for resistance independent of the selection for vine maturity classifications.

Marker-assisted selection (MAS) has been used in plant breeding but has lagged behind in certain crops such as potato (*Solanum tuberosum* ssp. *tuberosum*) because of complex genome structure, limiting linkage analysis to economically important traits. Cultivated autotetraploid potato (2*n* = 4*x* = 48) presents unique challenges for generating linkage maps. Population size and number of segregating markers required are both dictated by the complex nature of tetrasomic inheritance (Hackett *et al.* 1998). Comparatively few genetic markers are currently used in varietal potato breeding at the tetraploid level, probably resulting from the limited number of genetic analyses conducted at the tetraploid level. The recent development of the Infinium 8303 Potato Array has provided a genome-wide set of single-nucleotide polymorphism (SNP) markers that can be used in tetraploid mapping and quantitative trait locus (QTL) analysis (Hamilton *et al.* 2011; Felcher *et al.* 2012; Douches *et al.* 2014). Similarly, recent advances in the development of statistical models that incorporate allele dosage information (*i.e.*, the number of copies of each allele at a given polymorphic locus) have significantly increased the power of SNP data to detect recombination between loci and for interval mapping of QTL in autopolyploids (Hackett *et al.* 2013, 2014).

There is a great need to identify markers linked to disease resistance genes and agronomic traits in potato to apply MAS for cultivar development. Late blight of potato caused by *Phytophthora infestans* (Mont.) de Bary, is one of the most devastating diseases of cultivated potato. MAS can be one of the more efficient methods for the introgression of resistance into commercially desirable cultivars (Gebhardt *et al.* 2004). To date, numerous late blight resistance (*R*) genes/QTL have been identified in various *Solanum* species and used for MAS in cultivated potato (reviewed in Tiwari *et al.* 2013; Ramakrishnan *et al.* 2015). First introgressions were derived from the highly resistant hexaploid species *Solanum demissum*, which is the source of 11 single dominant *R* genes (Gebhardt *et al.* 2004). However, *R* gene-based qualitative resistance may provide only transient resistance against *P. infestans* infection. Previous works have shown that a more durable and greater level of resistance is achieved by pyramiding resistance through the introgression of multiple resistance genes (Nelson 1972). For instance, pyramiding *P. infestans* genes *R_Pi-mcd1_* and *R_Pi-ber_* in diploid *S. tuberosum* populations resulted in improved late blight resistance (Tan *et al.* 2010) compared with nontransformed cultivars. Similarly, a combination of at least five different *R* genes was shown to confer both qualitative and quantitative durable late blight resistant to the potato cultivar “Sarpo Mira” (Rietman *et al.* 2012). Therefore, late blight resistance genes/QTL identified through genetic mapping would be very useful in the introgression of resistance into commercial cultivars via marker-assisted pyramiding.

The primary aim of this research was to construct a genetic linkage map at the cultivated tetraploid level to locate QTL contributing to resistance to isolates of the US-8 genotype of *P. infestans*. Because of the strong evidence supporting the correlation between late blight resistance and lateness in plant maturity under long day conditions (Bormann *et al.* 2004), our study assessed plant maturity type to determine the linkage relationships among loci controlling both traits. The use of allele dosage information to generate the genetic maps in the tetraploid mapping population “Jacqueline Lee” × “MSG227-2” allowed the identification of major QTL for the traits. Candidate SNPs explaining most of the phenotypic variation could be linked to the potato reference genome. These SNPs are prime candidates for MAS for late blight resistance and vine maturity.

## Materials and Methods

### Plant material

The mapping population MSL603 consists of 156 F_1_ plants resulting from a cross between the cultivar “Jacqueline Lee” (Douches *et al.* 2001) the female parent and the MSU breeding line “MSG227-2” the male parent. “Jacqueline Lee” has an oval type tuber with light-yellow colored flesh, has general foliar resistance to a range of genotypes of *P. infestans*, and is susceptible to potato common scab (*Streptomyces scabies*) (Douches *et al.* 2001; Driscoll *et al.* 2009). The MSU-bred line “MSG227-2” (“Prestile” × “MSC127-3”) is resistant to potato common scab but susceptible to late blight (Driscoll *et al.* 2009).

### Phenotypic data

Potato late blight screening was conducted at MSU Muck Soils Research Farm, Bath, Michigan during the summers of 2002−2003. Each year, the plots were inoculated in late July with a zoospore suspension of the US-8 genotype through the irrigation system (Kirk *et al.* 2005). Three replications of the MSL603 population and parents were planted from tubers in early June in a randomized complete block design. Potato late blight severity was assessed by visually estimating percent coverage of foliar late blight lesions present in five-plant plots (Kirk *et al.* 2005; Young *et al.* 2009). Each plot was evaluated on at least seven occasions over approximately a 1-mo period after inoculation and completed when susceptible lines were 100% infected in both years of the study. The average of the relative area under the disease progress curve (RAUDPC) for each line (Kirk *et al.* 2005) was calculated and used for the QTL analyses. To assess late blight severity, the RAUDPC mean values were subjected to analysis of variance and then compared by the Scott-Knott clustering algorithm (Scott and Knott 1974) with an α value of 0.05, using the ‘ScottKnott’ R package (Jelihovschi *et al.* 2014).

Variance components of the RAUDPC scores from 2002 and 2003 were estimated by the restricted maximum likelihood method with years as fixed effects. Results were used to estimate broad sense heritability as follows:

$$H^{2}=\frac{\sigma_{g}^{2}}{\left(\sigma_{g}^{2}+\frac{\sigma_{g^{*}y}^{2}}{m}+\frac{\sigma_{e}^{2}}{rm}\right)}$$

where $\sigma_{g}^{2}$, $\sigma_{g^{*}y}^{2}$, and $\sigma_{e}^{2}$ are the genetic, genotype × year interaction, and residual variance components, *m* is the number of years, and *r* is the number of replications.

Ten-hill plots of the parents and progeny were planted at the MSU Montcalm Potato Research Center in 2002 and 2003 in a randomized complete block design. The study was planted May 5 and May 8 in 2002 and 2003, respectively. At 120 d after planting, vine maturity was visually scored on a 1−5 scale with 1 = early, all vines in a plot dead, while 5 = late, vigorous vines and flowering.

### SNP genotyping

DNA from the parents and progeny was extracted from freeze-dried leaf tissue using QIAGEN DNeasy Plant Mini Kits (QIAGEN, Germantown, MD), quantified with the Quant-iT PicoGreen assay (Invitrogen, San Diego, CA), and adjusted to a concentration of 50 ng·µL^−1^. Genome-wide SNP genotyping was performed with the Infinium 8303 Potato Array as described by Felcher *et al.* (2012). The Illumina GenomeStudio software (Illumina, Inc., San Diego, CA) was used to extract SNP theta scores for further analyses.

### Data processing

The SNP genomic positions are reported based on the PGSC Version 4.03 Pseudomolecules of the reference Potato *Solanum tuberosum* group Phureja DM1-3 516 R44 (Sharma *et al.* 2013). Only SNPs uniquely aligned to the reference genome were considered for further analysis. The Infinium 8303 Potato Array SNPs aligned to the PGSC Version 4.03 Pseudomolecules are available from Spud DB Web site (Hirsch *et al.* 2014).

Three filtering steps initially were applied to identify candidate SNPs suitable for further analysis as described (Hackett *et al.* 2013). First, SNPs were retained if the trimmed range between the 2% and 98% quantiles was equal or greater than 0.1. This step excluded monomorphic markers and removed any possible influence of outliers. Second, the theta values were modeled as a smooth function using locally weighted regression to remove SNPs showing a significant trend and/or differences between plates (with *P* < 0.0001) (Cleveland 1979; Hackett *et al.* 2013). Third, SNPs with missing theta values were removed.

### Genotype calling and allele dosage estimation

Five allele dosages are possible in a full-sib autotetraploid mapping population (AAAA, AAAB, AABB, ABBB, BBBB). To call genotypes using allele dosage information, we ran an application of a test version of TetraploidMap software (TPM, BioSS). This application fits mixture models to the theta scores distribution of the offspring and uses expected probabilities given the parental configurations as mixture proportions. It also provides the % variance of the theta score explained by configuration and shows maximum residuals to see if possible double reduction products are present (Hackett *et al.* 2013). As the analyses for linkage and QTL mapping assume random chromosomal segregation and no double reduction, SNPs with maximum residual values greater than 0.25 were inspected for potential double reduction products. Additional filtering steps were subsequently performed to exclude duplicates (cosegregating) and SNPs with >10% missing genotype calls.

### Linkage map construction and QTL analysis

Linkage map construction and QTL analysis followed the methodology described in Hackett *et al.* (2013) and Hackett *et al.* (2014). Steps for the construction of linkage maps included testing for distorted segregation, clustering SNPs, calculation of recombination fractions and LOD (logarithm of the odds) scores, ordering of SNPs, and inference of parental phase. An SNP marker was excluded as distorted if the significance of the χ^2^ goodness-of-fit statistic was less than 0.001 for simplex SNPs and 0.01 for duplex and greater dosage SNPs. For QTL mapping, we explored fitting a model of additive effects of each homolog. To establish the statistical significance for QTL, a permutation test was run with a minimum of 200 permutations and a 95% confidence interval for the LOD threshold was obtained as described by Nettleton and Doerge (2000). Subsequently simple models for the genotype means estimated at the most probable QTL position were explored, as described in Hackett *et al.* (2014). The best simple model was identified using the Schwarz Information Criterion (SIC) (Schwarz 1978) as follows:

$$SIC=-2\log L+p\log m_{o}$$

where *L* is the likelihood for the simple model, *p* is the number of parameters in the simple model, and *m_o_* is the number of observations (*i.e.*, the 36 genotype means). The best models are those with the lowest value for the SIC (Hackett *et al.* 2014). All steps were implemented with a test version of a TetraploidMap extension (C. Hackett, personal communication). MapChart 2.2 software was used for the graphical presentation of linkage maps and QTL (Voorrips 2002).

A high degree of concordance has been reported between linkage maps based on the Infinium 8303 Potato Array and the potato genome sequence (Felcher *et al.* 2012; Sharma *et al.* 2013). Here, we tested for concordance between the reference genome (PGSC Version 4.03 Pseudomolecules) and our linkage maps by comparing the genetic position (cM) with the physical location (Mb) of each SNP marker. This was performed using the R package MareyMap version 1.2 (Rezvoy *et al.* 2007). The plots were generated with the graphical interface MareyMapGUI and the slope of the curve was obtained using the “cubic splines” interpolation method.

### Data availability

The raw data collected and analyzed in this study is available in the supplemental files: Table S1 and Table S4. Additional details for the *Results and Discussion* are provided in the Supporting Information: Figure S1, Figure S2, Table S2, and Table S3.

## Results and Discussion

### SNP genotyping

A set of 7157 SNPs from the Infinium 8303 Potato Array maps to unique positions in the current PGSC Version 4.03 Pseudomolecules of the reference potato genome. After initial filtering to remove SNPs with missing theta values, and monomorphic markers, 3867 polymorphic SNPs remained for use in genotype calling. Nearly 95% of this set agrees with previously identified SNPs, from the Infinium 8303 Potato Array, having the ability to distinguish between the three heterozygous marker classes (AAAB, AABB, and ABBB) in 221 tetraploid potato lines (Hirsch *et al.* 2013). An additional 5% is reported here for MSL603. After genotype calling and further quality filtering (SNPs with double reduction, segregation distortion, and missing values), there were 1972 SNPs available for linkage analysis (Supporting Information, Table S1).

Markers with significant segregation distortion (*P* < 0.01) accounted for less than 6% of the 3867 segregating SNPs. Of the 145 distorted SNPs, 105 were duplex or higher dosage and 40 were simplex SNPs. Most of these SNPs were located on chromosomes 1, 2, 10, and 12 (Table 1). Clusters of distorted markers were observed on chromosomes 2 and 10. The distorted region on chromosome 2 (20 SNPs) spanned ∼8.5 Mb in the pericentromeric region, and the one on chromosome 10 (21 SNPs) was about 2 Mb length, close to a putative cluster of *Verticillium* wilt resistance genes on the long arm (PGSC Version 4.03). The remaining distorted markers were scattered throughout the chromosomes or in small groups spanning ≤1 Mb in length. In tetrasomic inheritance, double reduction is one of the major biological causes of segregation distortion (Luo *et al.* 2004). However, no major effect of double reduction was observed on loci with distorted segregation patterns, suggesting other causes, such as structural chromosome differences or selection for or against particular alleles or allelic combinations due to viability effects (Gebhardt *et al.* 1991).

**Table 1:** Summary of the parental linkage maps, “Jacqueline Lee” (JL) and “MSG227-2” (G227-2)

Chr	No. Mapped SNPs			Map Length, cM*^a^*		Map Length, Mb*^a^*		PGSC v4.03 PM, Mb*^a^*	Map Coverage*^a^*		Average Interloci Distance, cM		No. SNPs w/Segregation Distortion
	Total	JL	G227-2	JL	G227-2	JL	G227-2	DM	JL	G227-2	JL	G227-2	Total
1	187	140	131	117.7	117.4	88.1	87.5	88.7	0.99	0.99	0.85	0.90	27
2	183	147	126	86.3	86.7	43.4	43.2	48.6	0.89	0.89	0.59	0.69	23
3	166	134	106	95.5	85.7	62.0	57.2	62.3	1.00	0.82	0.72	0.82	8
4	192	142	125	85.3	84.5	69.9	69.9	72.2	0.97	0.97	0.61	0.68	2
5	167	104	140	87.6	87.6	51.9	51.9	52.1	1.00	1.00	0.85	0.63	2
6	173	139	115	83.7	88.9	57.6	58.3	59.5	0.97	0.98	0.61	0.78	8
7	162	128	126	86.1	86.4	55.3	55.3	56.8	0.97	0.97	0.68	0.69	4
8	151	102	105	76.6	77.2	56.4	56.4	56.9	0.99	0.99	0.76	0.74	1
9	153	118	114	90.8	81.8	61.3	57.4	61.5	1.00	0.93	0.78	0.72	9
10	138	99	101	90.9	88.9	59.3	59.3	59.8	0.99	0.99	0.93	0.89	33
11	152	116	108	86.8	93.6	44.6	44.6	45.5	0.98	0.98	0.75	0.88	3
12	148	110	128	85.1	89.6	60.4	60.6	61.2	0.99	0.99	0.78	0.71	25
Total	1972	1479	1425	1072.4	1068.3	712.8	708.5	725.1	0.98	0.96	0.74	0.76	145

SNP, single-nucleotide polymorphism.

a Map length (Mb) and map coverage values are based on the PGSC Version 4.03 Pseudomolecules of the reference potato *Solanum tuberosum* group Phureja DM1-3 516 R44 (DM).

Considering all parental genotypes configurations, simplex (AAAB × AAAA, ABBB × BBBB), duplex (AABB × AAAA, AABB × BBBB), and double-simplex (AAAB × AAAB, ABBB × ABBB) totaled 65% (1278), whereas 35% (694) were between triplex (AAAB × BBBB, ABBB × AAAA) and higher dosages (Table S2). Thus, with a large number of SNPs and a wide range of configurations, this dataset is likely to provide informative SNP pair combinations for the detection of linked loci and the estimation of recombination fractions (Hackett *et al.* 2013; Li *et al.* 2014). Moreover, with the incorporation of allele configurations that allow alignment of the parental maps (*i.e.*, shared SNP markers), allele effects from both parents can be studied simultaneously (Hackett *et al.* 2014).

### Linkage map construction and QTL analysis

**Figure 1:**
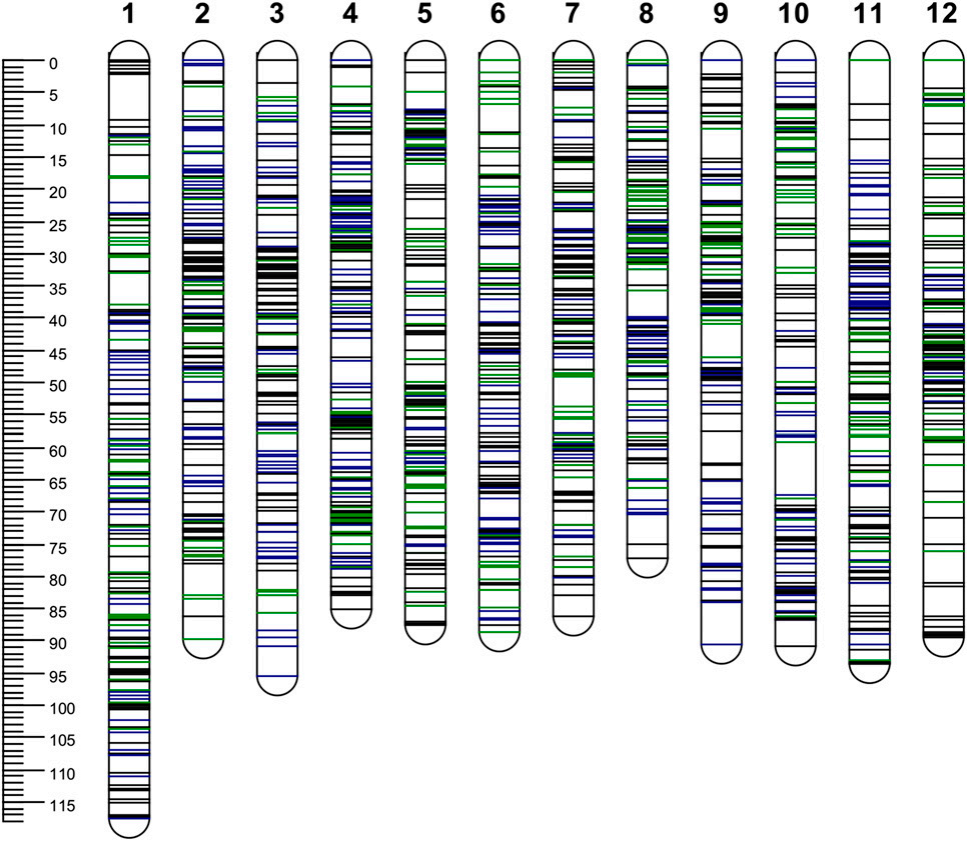
Distribution of single-nucleotide polymorphism (SNP) markers on 12 chromosomes (1−12). The scale shows the genetic distance in cM. SNPs positions are marked in dark blue (from “Jacqueline Lee”), green (from “MSG227-2”) and black (from both parents).

Of the 1972 SNPs available for linkage mapping, 1479 were segregating in “Jacqueline Lee” and 1425 in “MSG227-2,” with 932 SNPs segregating in both parents. Each of the parental maps spanned a genetic distance of 1072 and 1068 cM, ranging from 76.2 to 117.7 cM, with an average of 121 SNP markers per chromosome and a marker density of ∼1.36 SNP per cM. Both “Jacqueline Lee” and “MSG227-2” maps covered, on average, 98% of the PGSC v4.03 Pseudomolecules (Table 1, Table S3, and Figure 1).

### Late blight resistance

**Figure 2:**
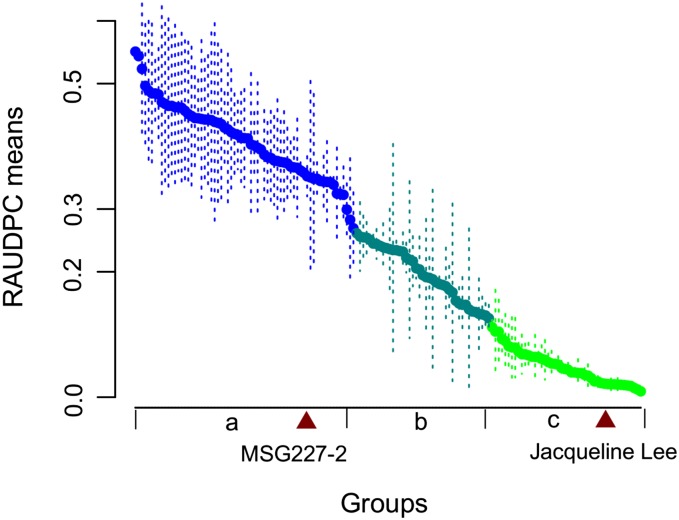
Genotype groups (a, b, c) for RAUDPC means as defined by the Scott-Knott algorithm (α = 0.05).

Plant response to foliar US-8 infection was consistent between the two years of observations (Table 2 and Table S4). The RAUDPC mean values obtained from 2002 and 2003 were significantly correlated with each other, with a Pearson’s correlation of 0.83 (*P* < 0.0001). Among the 156 progeny, 46 were classified as resistant (*i.e.*, RAUPDC means were not significantly different from that of the resistant parent “Jacqueline Lee”), 41 as intermediate, and 67 as susceptible (*i.e.*, RAUPDC means were not significantly different from that of the susceptible parent MSG227-2) by the Scott-Knott algorithm (Figure 2). Two clones were not included in the analysis because of missing data. The broad-sense heritability (*H^2^*) for both years showed statistically significant consistency, with a combined *H^2^* estimate of 0.86. These results indicate a substantial genetic effect and suggest major gene effects for the trait.

**Table 2:** Parents and population late blight (RAUDPC) and maturity (MAT) mean, SD, minimum (min), and maximum (max) trait values for 2 yr

Trait	Year	MSG227-2	J. Lee	F1 Progeny			
				Mean	SD	Min	Max
RAUDPC_2	2002	0.34	0.04	0.21	0.13	0.01	0.43
RAUDPC_3	2003	0.36	0.01	0.27	0.20	0.01	0.63
MAT_2	2002	2.72	2.00	1.72	0.83	1.00	5.20
MAT_3	2003	3.75	2.75	2.58	1.05	1.00	4.75

RAUDPC, relative area under the disease progress curve.

**Figure 3:**
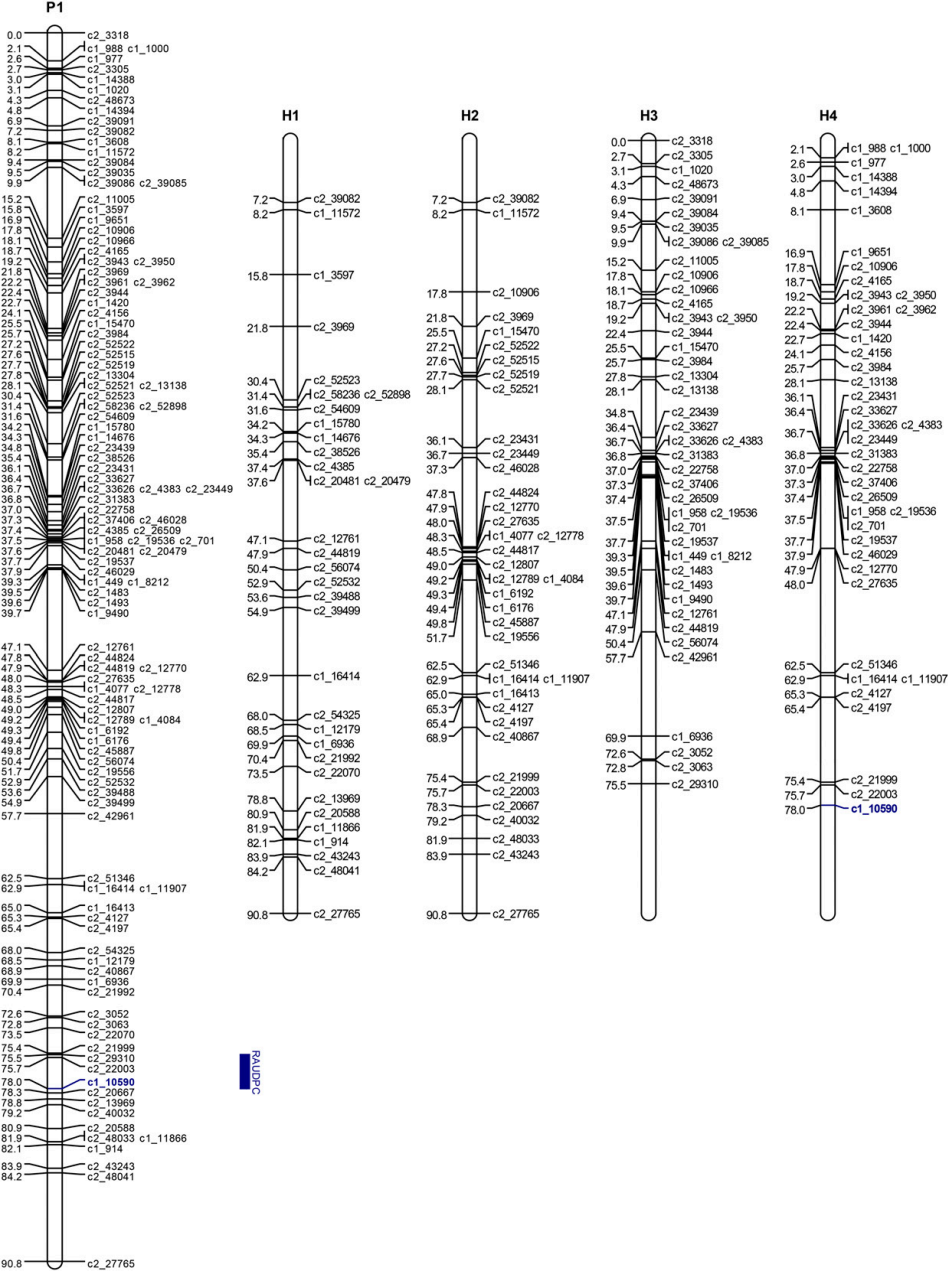
Linkage map of “Jacqueline Lee” chromosome 9. P1: overall map. H1−H4: homologous maps. The blue bar corresponds to the two-LOD support interval for the QTL location.

When the RAUDPC values were mapped to the linkage maps, the most significant association was detected on chromosome 9. For year 2002, this association had a maximum LOD of 22.1 and explained 39.3% of the trait variance, and for 2003 the maximum LOD was 14.2 and it explained 25.7% of the phenotypic variance (Table 3). These LOD scores well were above the upper permutation thresholds of 4.43 and 4.75 for 2002 and 2003, respectively. The peak LOD for both years was at 78.0 cM. Examination of different simple genetic models indicated that the best-fitting model was for “Jacqueline Lee,” having a simplex allele AAAB with the B allele associated with increase in resistance (*P* < 0.001) on homologous chromosome 4 (H4, Figure 3). This model had the lowest SIC of −239.0 and −191.2 for year 2002 and 2003, respectively, compared to the full model with SIC = −217.6 and −171.3, respectively. The closest SNP with this configuration is the simplex SNP c1_10590 at 78.02 cM. Regression of the RAUDPC values on the genotype of this SNP explained 63.3% of the phenotypic variance for 2002 and 45.0% for 2003. The B allele was present in 44 of the 46 resistant clones and absent in 62 of the 67 susceptible clones.

**Table 3:** QTL location from analysis without iteration for the RAUDPC trait scored on the MSL603 population during 2002−2003

Trait	Chromosome	LOD	LOD threshold 95% CI	No. Permutations	*R^2^*	Model, Homologous Chromosome, and Candidate SNPs
Late Blight -2002	9	22.1	3.52−4.43	200	39.3	Simplex, h4, c1_10590
Late Blight -2003	9	14.2	3.61−4.75	200	25.7	Simplex, h4, c1_10590
Maturity-2002	5	7.2	3.43−4.49	200	14.7	Duplex-simplex, c2_22986
Maturity-2003	5	8.3	3.52−4.48	200	16.1	Duplex-simplex, c2_22986

QTL, quantitative trait loci; RAUDPC, relative area under the disease progress curve; LOD, logarithm of the odds; CI, confidence interval; SNP, single-nucleotide polymorphism.

The SNP solcap_snp_c1_10590 maps to the distal end of the long arm of chromosome 9, at position 60.3 Mb of the PGSC v4.03 Pseudomolecules (superscaffold PGSC0003DMB000000280) and is ∼0.54 Mb distal to a late blight resistance-hotspot region on the adjacent superscaffold PGSC0003DMB000000339. This region harbors Toll/Interleukin-1 Receptor (TIR)-Nucleotide Binding Site (NBS)-Leucine-Rich Repeat (LRR) genes and close homologs of the *Rpi-vnt1* gene from *Solanum venturii* (PGSC version 4.03). The hotspot for resistance on the long arm of chromosome 9 is a relatively conserved region among *Solanum* species (Pel *et al.* 2009). In addition to TIR-NBS-LRR and *Rpi-vnt1*-like (NB-LRR) genes, the region harbors a cluster of close homologs of the Tospovirus resistance gene *Sw-5* at the distal end (Foster *et al.* 2009; Jupe *et al.* 2012; Smilde *et al.* 2005; Zhang *et al.* 2014). Collectively, these results make the SNP solcap_snp_c1_10590 a prime candidate for MAS.

The source of foliar resistance to *P. infestans* in “Jacqueline Lee,” the cultivar “Tollocan”, was derived from the hexaploid Mexican species *S. demissum* and possibly from local varieties such as “Amarilla de Puebla” and “Leona” (Grunwald *et al.* 2002). However, the *R* gene-based late blight resistance in “Jacqueline Lee” had not been determined (Douches *et al.* 2001). *Solanum demissum* is the source of 11 single dominant late blight resistance genes (*R1-R11*) (Huang *et al.* 2005; Bradshaw *et al.* 2006; Hein *et al.* 2009; Jo *et al.* 2011; Vleeshouwers *et al.* 2011). Two of these *R* genes, *R8* and *R9* (*R9a*, Jo *et al.* 2015), have been mapped recently to the long arm of chromosome 9, within the resistance hotspot (Jo *et al.* 2011; Vossen *et al.* 2014; Jo *et al.* 2015).

**Figure 4:**
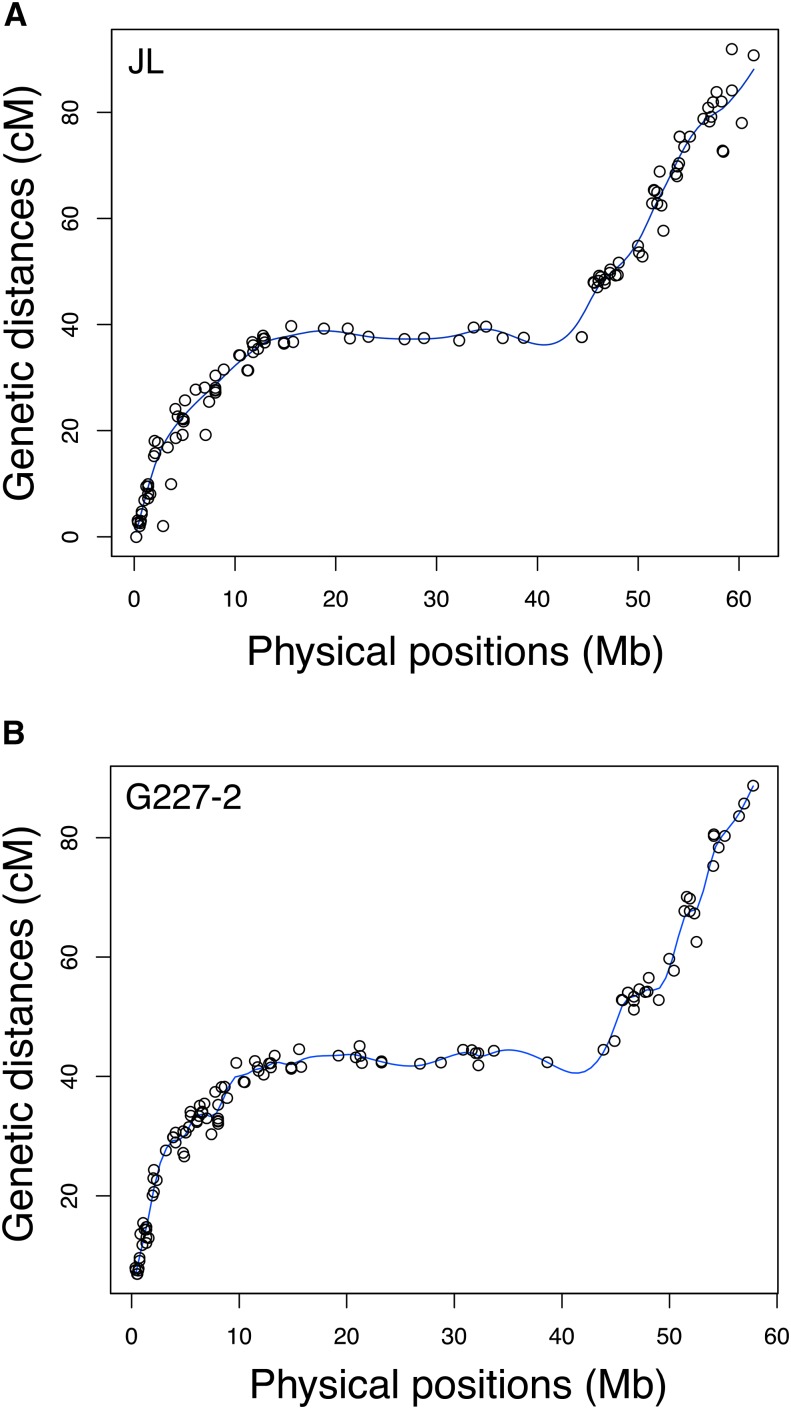
Graph of chromosome 9 showing the genetic location (cM) and the physical position (Mb) of SNP markers. (A)“Jacqueline Lee” (JL), 118 markers. (B) “MSG227-2” (G227-2), 114 markers.

By comparing the genetic location (cM) with the physical position (Mb) of each marker on chromosome 9, we assessed the concordance between the genetic and physical maps. The resulting graphs displayed the expected shape and fit well with chromosome structure (Figure 4) (Felcher *et al.* 2012; Sharma *et al.* 2013). Thus, the detection of a major effect QTL for late blight resistance on the long arm of chromosome 9 of “Jacqueline Lee,” along with the identification of a candidate SNP (solcap_snp_c1_10590) associated to this QTL, provides strong evidence for the location of genes controlling the trait. Whether a single gene or multiple genes from this region are responsible for late blight resistance requires further investigation. The phenotypic resistance in the MSL603 population departed from a 1:1 segregation, with a bias toward susceptibility. A single gene with strong environmental effects, multiple *R*-genes with both qualitative and quantitative effects, conditional QTL (Li *et al.* 2012), or other genetic factors underlying the trait could explain this segregation pattern. In addition to the major QTL on chromosome 9, the probability of a minor effect QTL on the distal end of chromosome 11, close to a cluster of NB-LRR genes (Jupe *et al.* 2012) cannot be ruled out. The occurrence of this minor effect QTL however needs to be confirmed, as only one year (2002) was statistically supported (data not shown).

### Maturity

“Jacqueline Lee” is a selection from a cross between the late-maturing Mexican variety “Tollocan” and the early-maturing variety “Chaleur” (De Jong *et al.* 1995) with mid-season maturity (Douches *et al.* 2001). In this study both parents showed similar phenotypic scores ranging from early mid-season to mid-season maturity. The population mean was slightly lower than the two parents, whereas the population trait values showed a wide range of phenotypic variation (Table 2 and Table S4). When the mean values were mapped, a significant association was found on chromosome 5. For year 2002, a LOD of 7.2 explained 14.7% of the phenotypic variance and for 2003, a LOD of 8.3 explained 16.1% of the trait variance. These LOD scores were above the upper bound of the permutation thresholds of 4.38 and 4.39 for 2002 and 2003, respectively. The peak LOD was detected at 21.0 cM in both years (Table 3). Exploration of different genetic models indicated that there is not a simple model explaining the trait variance. However, there is a possibility for a duplex-simplex configuration with a duplex marker on “Jacqueline Lee” and a simplex marker on “MSG227-2”, suggesting a QTL with allele effects from both parents (Figure S1). The closest SNP with a duplex-simplex configuration (BBAA × BAAA) is the SNP solcap_snp_c2_22986 at 20.6 cM with the B allele associated with increase in lateness (*P* < 0.0001). Thus, progeny with two or three copies of the B allele (AABB, ABBB) showed on average later maturity than progeny with none or a single copy (AAAA, AAAB). Regression of the maturity values on the genotype of this SNP further explained 19.0% of the phenotypic variance for 2002 and 16.0% for 2003.

The SNP solcap_snp_c2_22986 maps to the distal end of the short arm of chromosome 5 at position 4.28 Mb on the *ATP-dependent helicase* gene (PGSC0003DMG402030493) of the reference Potato (*Solanum tuberosum* group Phureja DM1-3 516 R44) PGSC v4.03 Pseudomolecules. This SNP (solcap_snp_c2_22986) is ∼0.25 Mb from the *StCDF1* (*Solanum tuberosum* CDF gene 1, PGSC0003DMG400018408) gene, which is a candidate gene for the plant maturity phenotype in potato (Kloosterman *et al.* 2013). Similar to what was observed in chromosome 9, the comparison of the genetic location (cM) with the physical position (Mb) of each marker on chromosome 5 showed a high degree of concordance (Figure S2). Combined, these results provide strong evidence for the location of genes controlling the trait. Further validation of solcap_snp_c2_22986 could potentially lead to a diagnostic marker for maturity.

The present study reports for the first time the major source of late blight resistance in “Jacqueline Lee.” This cultivar is a unique source of resistance to *P. infestans*. It has exhibited consistently high levels of resistance not only to US-8 (Douches *et al.* 2001; Douches *et al.* 2004; Young *et al.* 2009) but also to US-22 and US-23, which have affected Michigan and the Northeastern United States (Rojas *et al.* 2014). With the availability of a genome-wide set of genetic markers and the recently developed statistical tools for genetic linkage analysis and QTL mapping that incorporate allele dosage information, we were able to identify a major-effect QTL and a candidate SNP marker associated with a decrease in late blight infection. With this information in place, the phenotypic variation for the trait can be linked directly to the potato reference genome. Thus, the location of the candidate SNP, close to the late blight resistant hotspot on chromosome 9, provides a strong statistical support for the NB-LRR gene-based resistance. This study also reports a major-effect QTL and a SNP marker associated to plant maturity on chromosome 5. The presence of this QTL not linked to the QTL for late blight resistance offers the possibility of using MAS for resistance independent of the selection for plant maturity.

## 

## Supplementary Material

Supporting Information

## References

[bib1] Bormann C. A. Rickert A. M. Castillo Ruiz R. A. Paal J. Lübeck J., 2004 Tagging quantitative trait loci for maturity-corrected late blight resistance in tetraploid potato with PCR-based candidate gene markers. Mol. Plant Microbe Interact. 17: 1126–1138. 15497405 10.1094/MPMI.2004.17.10.1126

[bib2] Bradshaw J. Bryan G. Lees A. McLean K. Solomon-Blackburn R., 2006 Mapping the *R10* and *R11* genes for resistance to late blight (*Phytophthora infestans*) present in the potato (*Solanum tuberosum*) R-gene differentials of Black. Theor. Appl. Genet. 112: 744–751. 16395567 10.1007/s00122-005-0179-9

[bib3] Cleveland W. S., 1979 Robust locally weighted regression and smoothing scatterplots. J. Am. Stat. Assoc. 74: 829–836.

[bib4] De Jong H. Tarn T. R. Murphy A. M. Tai G. C. C. Bagnall R. H., 1995 Ac chaleur: a new early-maturing cultivar with excellent table quality. Am. Potato J. 72: 393–399.

[bib5] Douches D. Coombs J. Felcher K. Kirk W., 2004 Foliar reaction to *Phytophthora infestans* in inoculated potato field trials in Michigan. Am. J. Potato Res. 81: 443–448.

[bib6] Douches D. Hirsch C. N. Manrique-Carpintero N. C. Massa A. N. Coombs J., 2014 The contribution of the solanaceae coordinated agricultural project to potato breeding. Potato Res. 57: 215–224.

[bib7] Douches D. S. Jastrzebski K. Coombs J. Kirk W. W. Felcher K. K., 2001 Jacqueline Lee: a late-blight-resistant tablestock variety. Am. J. Potato Res. 78: 413–419.

[bib8] Driscoll J. Coombs J. Hammerschmidt R. Kirk W. Wanner L., 2009 Greenhouse and field nursery evaluation for potato common scab tolerance in a tetraploid population. Am. J. Potato Res. 86: 96–101.

[bib9] Felcher K. J. Coombs J. J. Massa A. N. Hansey C. N. Hamilton J. P., 2012 Integration of two diploid potato linkage maps with the potato genome sequence. PLoS One 7: e36347. 22558443 10.1371/journal.pone.0036347 PMC3338666

[bib10] Foster S. Park T. Pel M. Brigneti G. Sliwka J., 2009 *Rpi-vnt1.1*, a *Tm-22* homolog from *Solanum venturii*, confers resistance to potato late blight. Mol. Plant Microbe Interact. 22: 589–600. 19348576 10.1094/MPMI-22-5-0589

[bib11] Gebhardt C. Ritter E. Barone A. Debener T. Walkemeier B., 1991 RFLP maps of potato and their alignment with the homoeologous tomato genome. Theor. Appl. Genet. 83: 49–57. 24202256 10.1007/BF00229225

[bib12] Gebhardt C. Ballvora A. Walkemeier B. Oberhagemann P. Schüler K., 2004 Assessing genetic potential in germplasm collections of crop plants by marker-trait association: a case study for potatoes with quantitative variation of resistance to late blight and maturity type. Mol. Breed. 13: 93–102.

[bib13] Grunwald N. J. Cadena Hinojosa M. A. Covarrubias O. R. Pena A. R. Niederhauser J. S., 2002 Potato cultivars from the Mexican National Program: sources and durability of resistance against late blight. Phytopathology 92: 688–693. 18943263 10.1094/PHYTO.2002.92.7.688

[bib14] Hackett C. Bradshaw J. Bryan G., 2014 QTL mapping in autotetraploids using SNP dosage information. Theor. Appl. Genet. 127: 1885–1904. 24981609 10.1007/s00122-014-2347-2 PMC4145212

[bib15] Hackett C. A. Bradshaw J. E. Meyer R. C. McNicol J. W. Milbourne D., 1998 Linkage analysis in tetraploid species: a simulation study. Genet. Res. 71: 143–153.

[bib16] Hackett C. A. McLean K. Bryan G. J., 2013 Linkage analysis and QTL mapping using SNP dosage data in a tetraploid potato mapping population. PLoS One 8: e63939. 23704960 10.1371/journal.pone.0063939 PMC3660524

[bib17] Hamilton J. Hansey C. Whitty B. Stoffel K. Massa A., 2011 Single nucleotide polymorphism discovery in elite north american potato germplasm. BMC Genomics 12: 302. 21658273 10.1186/1471-2164-12-302 PMC3128068

[bib18] Hein I. Birch P. J. Danan S. Lefebvre V. Achieng Odeny D., 2009 Progress in mapping and cloning qualitative and quantitative resistance against *Phytophthora infestans* in potato and its wild relatives. Potato Res. 52: 215–227.

[bib19] Hirsch C. D. Hamilton J. P. Childs K. L. Cepela J. Crisovan E., 2014 Spud DB: A resource for mining sequences, genotypes, and phenotypes to accelerate potato breeding. Plant Genome 7: 1–12.

[bib20] Hirsch C. N. Hirsch C. D. Felcher K. Coombs J. Zarka D., 2013 Retrospective view of North American potato (Solanum tuberosum L.) breeding in the 20th and 21st centuries. G3 (Bethesda) 3: 1003–1013. 23589519 10.1534/g3.113.005595 PMC3689798

[bib21] Huang S. Van Der Vossen E. A. G. Kuang H. Vleeshouwers V. G. A. A. Zhang N., 2005 Comparative genomics enabled the isolation of the *R3a* late blight resistance gene in potato. Plant J. 42: 251–261. 15807786 10.1111/j.1365-313X.2005.02365.x

[bib22] Jelihovschi E. G. Faria J. C. Bezerra Allaman I., 2014 ScottKnott: a package for performing the Scott-Knott clustering algorithm in R. TEMA 15: 3–7.

[bib23] Jo K. R. Arens M. Kim T. Y. Jongsma M. Visser R. F., 2011 Mapping of the *S. demissum* late blight resistance gene *R8* to a new locus on chromosome IX. Theor. Appl. Genet. 123: 1331–1340. 21877150 10.1007/s00122-011-1670-0 PMC3214258

[bib24] Jo K. R. Visser R. F. Jacobsen E. Vossen J., 2015 Characterisation of the late blight resistance in potato differential *MaR9* reveals a qualitative resistance gene, *R9a*, residing in a cluster of *Tm-2* (2) homologs on chromosome IX. Theor. Appl. Genet. 128: 931–941. 25725999 10.1007/s00122-015-2480-6 PMC4544503

[bib25] Jupe F. Pritchard L. Etherington G. MacKenzie K. Cock P., 2012 Identification and localisation of the NB-LRR gene family within the potato genome. BMC Genomics 13: 75. 22336098 10.1186/1471-2164-13-75 PMC3297505

[bib26] Kirk W. W. Abu-El Samen F. M. Muhinyuza J. B. Hammerschmidt R. Douches D. S., 2005 Evaluation of potato late blight management utilizing host plant resistance and reduced rates and frequencies of fungicide applications. Crop Prot. 24: 961–970.

[bib27] Kloosterman B. Abelenda J. A. Carretero-Gomez M. M. Oortwijn M. de Boer J. M., 2013 Naturally occurring allele diversity allows potato cultivation in northern latitudes. Nature 495: 246–250. 23467094 10.1038/nature11912

[bib28] Li J. Lindqvist-Kreuze H. Tian Z. Liu J. Song B., 2012 Conditional QTL underlying resistance to late blight in a diploid potato population. Theor. Appl. Genet. 124: 1339–1350. 22274766 10.1007/s00122-012-1791-0

[bib29] Li X. Wei Y. Acharya A. Jiang Q. Kang J., 2014 A saturated genetic linkage map of autotetraploid alfalfa (Medicago sativa L.) developed using genotyping-by-sequencing is highly syntenous with the Medicago truncatula genome. G3 (Bethesda) 4: 1971–1979. 25147192 10.1534/g3.114.012245 PMC4199703

[bib30] Luo Z. W. Zhang R. M. Kearsey M. J., 2004 Theoretical basis for genetic linkage analysis in autotetraploid species. Proc. Natl. Acad. Sci. USA 101: 7040–7045. 15100415 10.1073/pnas.0304482101 PMC406462

[bib31] Nelson R. R., 1972 Stabilizing racial populations of plant pathogens by use of resistance genes. J. Environ. Qual. 1: 220–227.

[bib32] Nettleton D. Doerge R. W., 2000 Accounting for variability in the use of permutation testing to detect quantitative trait loci. Biometrics 56: 52–58. 10783776 10.1111/j.0006-341x.2000.00052.x

[bib33] Pel M. A. Foster S. J. Park T. H. Rietman H. van Arkel G., 2009 Mapping and cloning of late blight resistance genes from *Solanum venturii* using an interspecific candidate gene approach. Mol. Plant Microbe Interact. 22: 601–615. 19348577 10.1094/MPMI-22-5-0601

[bib34] Ramakrishnan A. Ritland C. Blas Sevillano R. Riseman A., 2015 Review of potato molecular markers to enhance trait selection. Am. J. Pot Res. 92: 455–472.

[bib35] Rezvoy C. Charif D. Gueguen L. Marais G. A. B., 2007 MareyMap: an R-based tool with graphical interface for estimating recombination rates. Bioinformatics 23: 2188–2189. 17586550 10.1093/bioinformatics/btm315

[bib36] Rietman H. Bijsterbosch G. Cano L. M. Lee H. R. Vossen J. H., 2012 Qualitative and quantitative late blight resistance in the potato cultivar Sarpo Mira is determined by the perception of five distinct RXLR effectors. Mol. Plant Microbe Interact. 25: 910–919. 22414442 10.1094/MPMI-01-12-0010-R

[bib37] Rojas J. A. Kirk W. W. Gachango E. Douches D. S. Hanson L. E., 2014 Tuber blight development in potato cultivars in response to different genotypes of *Phytophthora infestans*. J. Phytopathol. 162: 33–42.

[bib38] Schwarz G., 1978 Estimating the dimension of a model. Ann. Stat. 6: 461–464.

[bib39] Scott A. J. Knott M., 1974 A cluster analysis method for grouping means in the analysis of variance. Biometrics 30: 507–512.

[bib40] Sharma S. K. Bolser D. de Boer J. Sønderkær M. Amoros W., 2013 Construction of reference chromosome-scale pseudomolecules for potato: Integrating the potato genome with genetic and physical maps. G3 (Bethesda) 3: 2031–2047. 24062527 10.1534/g3.113.007153 PMC3815063

[bib41] Smilde W. D. Brigneti G. Jagger L. Perkins S. Jones J. D. G., 2005 *Solanum mochiquense* chromosome IX carries a novel late blight resistance gene *Rpi-moc1*. Theor. Appl. Genet. 110: 252–258. 15672258 10.1007/s00122-004-1820-8

[bib42] Tan M. Y. A. Hutten R. C. B. Visser R. G. F. van Eck H. J., 2010 The effect of pyramiding *Phytophthora infestans* resistance genes R *Pi-mcd1* and R *Pi-ber* in potato. Theor. Appl. Genet. 121: 117–125. 20204320 10.1007/s00122-010-1295-8 PMC2871099

[bib43] Tiwari J. K. Siddappa S. Singh B. P. Kaushik S. K. Chakrabarti S. K., 2013 Molecular markers for late blight resistance breeding of potato: an update. Plant Breed. 132: 237–245.

[bib44] Vleeshouwers V. G. A. A. Raffaele S. Vossen J. H. Champouret N. Oliva R., 2011 Understanding and exploiting late blight resistance in the age of effectors. Annu. Rev. Phytopathol. 49: 507–531. 21663437 10.1146/annurev-phyto-072910-095326

[bib45] Voorrips R. E., 2002 MapChart: software for the graphical presentation of linkage maps and QTLs. J. Hered. 93: 77–78. 12011185 10.1093/jhered/93.1.77

[bib46] Vossen J. H. Jo K. R. Vosman B., 2014 Mining the genus Solanum for increasing disease resistance, pp. 27–46 in Genomics of Plant Genetic Resources, edited by Tuberosa R. Frison E. Graner A. Springer, Dordrecht.

[bib47] Young G. K. Cooke L. R. Kirk W. W. Tumbalam P. Perez F. M., 2009 Influence of competition and host plant resistance on selection in *Phytophthora infestans* populations in Michigan, USA and in Northern Ireland. Plant Pathol. 58: 703–714.

[bib48] Zhang C. Liu L. Wang X. Vossen J. Li G., 2014 The *Ph-3* gene from *Solanum pimpinellifolium* encodes CC-NBS-LRR protein conferring resistance to *Phytophthora infestans*. Theor. Appl. Genet. 127: 1353–1364. 24756242 10.1007/s00122-014-2303-1 PMC4035550

