# Supplementary material for: Genetic Linkage Mapping of Economically Important Traits in Cultivated Tetraploid Potato (Solanum tuberosum L.)
Source: G3 (Bethesda). 2015 Sep 14;5(11):2357–64. doi: 10.1534/g3.115.019646 (PMC4632055; doi:10.1534/g3.115.019646)
Supplement: Supporting Information [file supp_5_11_2357__index.html]

Genetic Linkage Mapping of Economically Important Traits in Cultivated Tetraploid Potato (Solanum tuberosum L.) — Supporting Information 

# Genetic Linkage Mapping of Economically Important Traits in Cultivated Tetraploid Potato (*Solanum tuberosum* L.)

## Supporting Information for Massa *et al.*, 2015

**Files in this Data Supplement:**

- Supporting Information - Figures S1-S2 and Tables S1-S4 (PDF, 258 KB)
- Figure S1 - Linkage maps of "Jacqueline Lee" (P1) and "MSG227-2" (P2) chromosome 5. (PDF, 163 KB)
- Figure S2 - Graph of chromosome 5 showing the genetic location (cM) and the physical position (Mb) of SNP markers. (PDF, 189 KB)
- Table S2 - Parent and F1 offspring genotype configurations in the MSL603 tetraploid mapping population. (PDF, 150 KB)
- Table S1 - Genotype scores of the Infinium 8303 Potato Array SNP markers (AAAA=0, AAAB=1, AABB=2, ABBB=3, BBBB=4, and missing value=.) evaluated in the MSL603 population. (.txt, 650 KB)
- Table S3 - Order of the mapped SNPs on chromosomes 1-12. SNPs positions are marked with JL (from Jacqueline Lee), G227-2 (from MSG227-2), and blank (from both parents). (.txt, 57 KB)
- Table S4 - Parents and population late blight (RAUDPC) and maturity (MAT) raw/clonal phenotypic trait data. (.txt, 14 KB)
