# Supplementary material for: Genetic Linkage Mapping of Economically Important Traits in Cultivated Tetraploid Potato (Solanum tuberosum L.)
Source: G3 (Bethesda). 2015 Sep 14;5(11):2357–64. doi: 10.1534/g3.115.019646 (PMC4632055; doi:10.1534/g3.115.019646)
Supplement: Supporting Information [file supp_g3.115.019646_019646SI.pdf]

G3 Genes | Genomes | Genetics

Genetic Linkage Mapping of Economically Important Traits in Cultivated Tetraploid Potato (*Solanum tuberosum* L.)

Alicia N. Massa[*], Norma C. Manrique-Carpintero[*], Joseph J. Coombs[*], Daniel G. Zarka[*], Anne E. Boone[*], William W. Kirk[*], Christine A. Hackett[‡], Glenn J. Bryan[§], and David S. Douches[*,1]

[*]Department of Plant, Soil and Microbial Sciences, Michigan State University, East Lansing, Michigan 48824

[‡]Biomathematics and Statistics Scotland, Invergowrie, Dundee, DD2 5DA, UK

[§]The James Hutton Institute, Invergowrie, Dundee, DD2 5DA, UK

[1]Corresponding author: Department of Plant, Soil and Microbial Sciences, Michigan State University, East Lansing, MI 48824. E-mail: douchesd@msu.edu

DOI: 10.1534/g3.115.019646

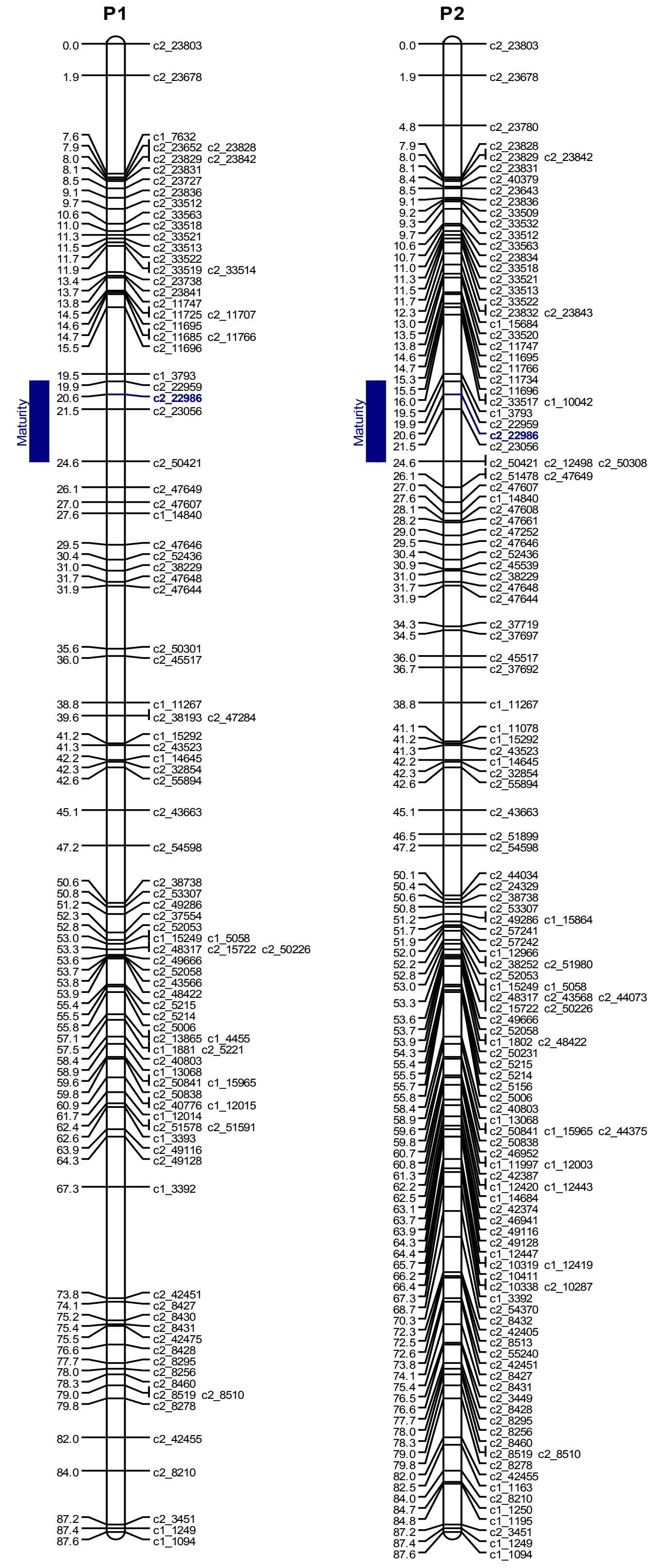

Figure S1 Linkage maps of “Jacqueline Lee” (P1) and “MSG227-2” (P2) chromosome 5.

The blue bar corresponds to the two-LOD support interval for the QTL location.

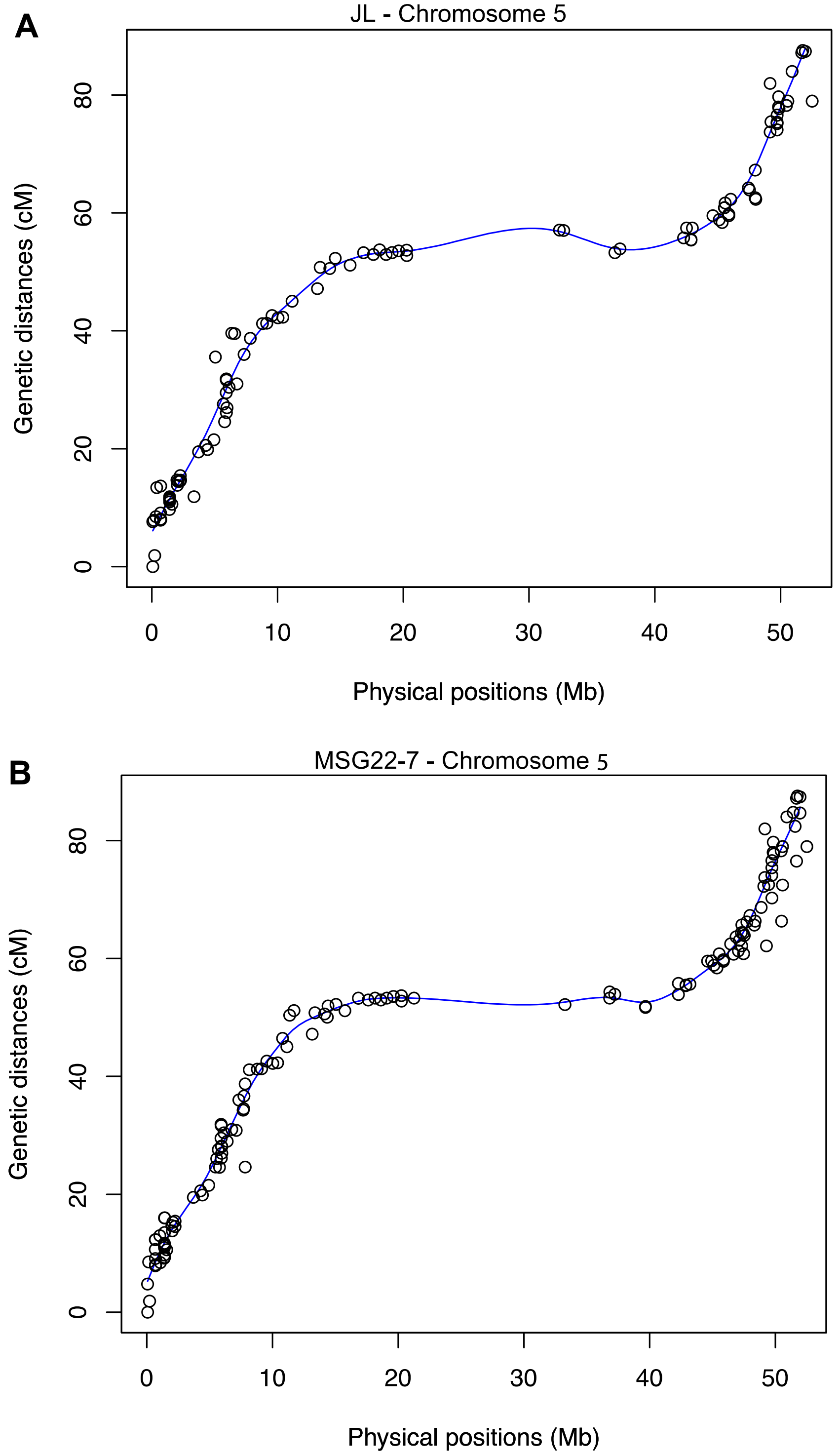

Figure S2 Graph of chromosome 5 showing the genetic location (cM) and the physical position (Mb) of SNP markers. A: "Jacqueline Lee" (JL), 104 markers. B: "MSG227-2", 140 markers.

Table S1 Genotype scores of the Infinium 8303 Potato Array SNP markers (AAAA=0, AAAB=1, AABB=2, ABBB=3, BBBB=4, and missing value=.) evaluated in the MSL603 population.

Available for download as a .txt file at www.g3journal.org/lookup/suppl/doi:10.1534/g3.115.019646/-/DC1

Table S2 Parent and F1 offspring genotype configurations in the MSL603 tetraploid mapping population.

No. Clusters	Parental genotype configurations		Type	Counts
2	AAAA	AAAB	Simplex	351
	BBBB	ABBB	Simplex	352
	AAAA	ABBB	Triplex	36
	AAAB	BBBB	Triplex	28
3	AAAA	AABB	Duplex	124
	AABB	BBBB	Duplex	149
	AAAB	AAAB	Double-simplex	143
	ABBB	ABBB	Double-simplex	159
	AAAB	ABBB	X-double-simplex	59
4	AAAB	AABB	Simplex-duplex	182
	AABB	ABBB	Duplex-simplex	254
5	AABB	AABB	Double-duplex	135
**Total**				**1972**

Tables S3-S4
Available for download as .txt files at
www.g3journal.org/lookup/suppl/doi:10.1534/g3.115.019646/-/DC1

Table S3. Order of the mapped SNPs on chromosomes 1-12. SNPs positions are marked with JL (from Jacqueline Lee), G227-2 (from MSG227-2), and blank (from both parents).

Table S4. Parents and population late blight (RAUDPC) and maturity (MAT) raw/clonal phenotypic trait data.
