# Supplementary material for: Genetic Linkage Mapping of Economically Important Traits in Cultivated Tetraploid Potato (Solanum tuberosum L.)
Source: G3 (Bethesda). 2015 Sep 14;5(11):2357–64. doi: 10.1534/g3.115.019646 (PMC4632055; doi:10.1534/g3.115.019646)
Supplement: Supporting Information [file supp_g3.115.019646_FigureS1.pdf]

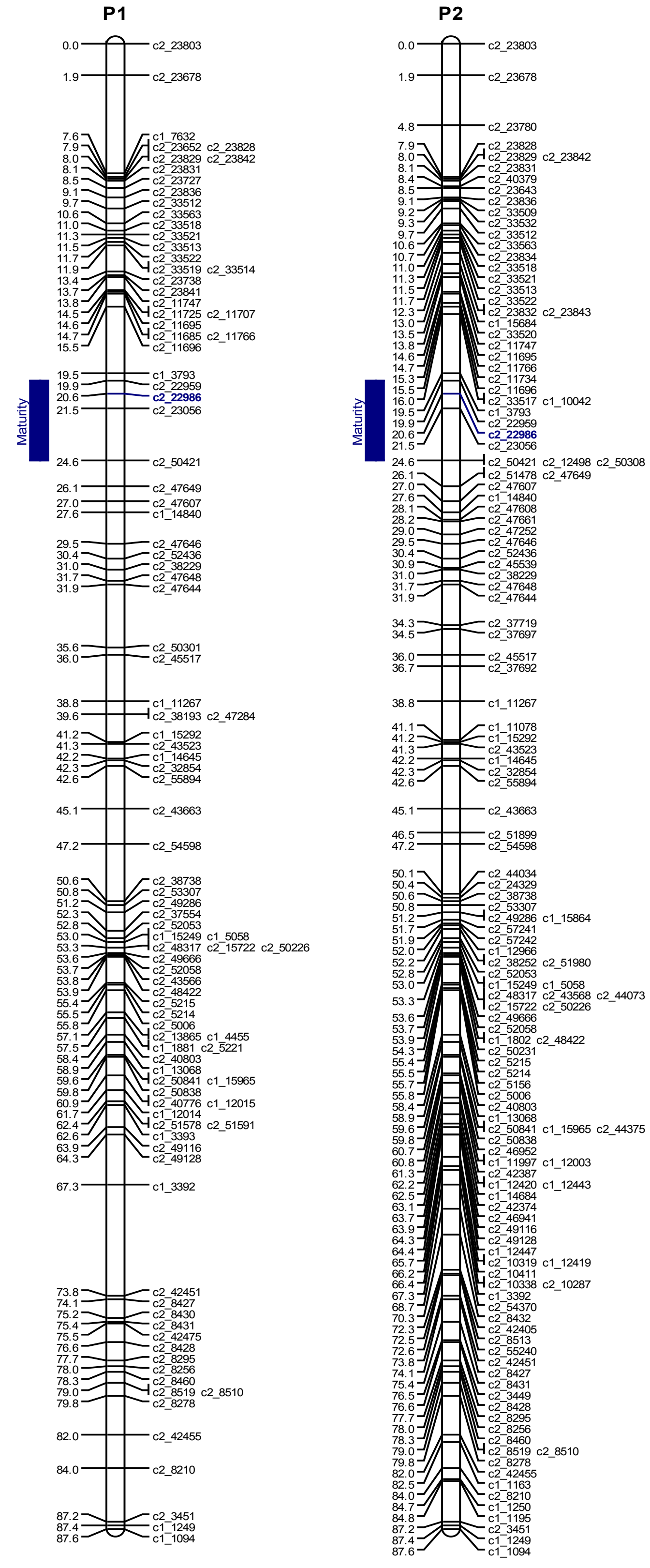

Figure S1 Linkage maps of "Jacqueline Lee" (P1) and "MSG227-2" (P2) chromosome 5.

The blue bar corresponds to the two-LOD support interval for the QTL location.
