# Supplementary material for: Genetic Linkage Mapping of Economically Important Traits in Cultivated Tetraploid Potato (Solanum tuberosum L.)
Source: G3 (Bethesda). 2015 Sep 14;5(11):2357–64. doi: 10.1534/g3.115.019646 (PMC4632055; doi:10.1534/g3.115.019646)
Supplement: Supporting Information [file supp_g3.115.019646_FigureS2.pdf]

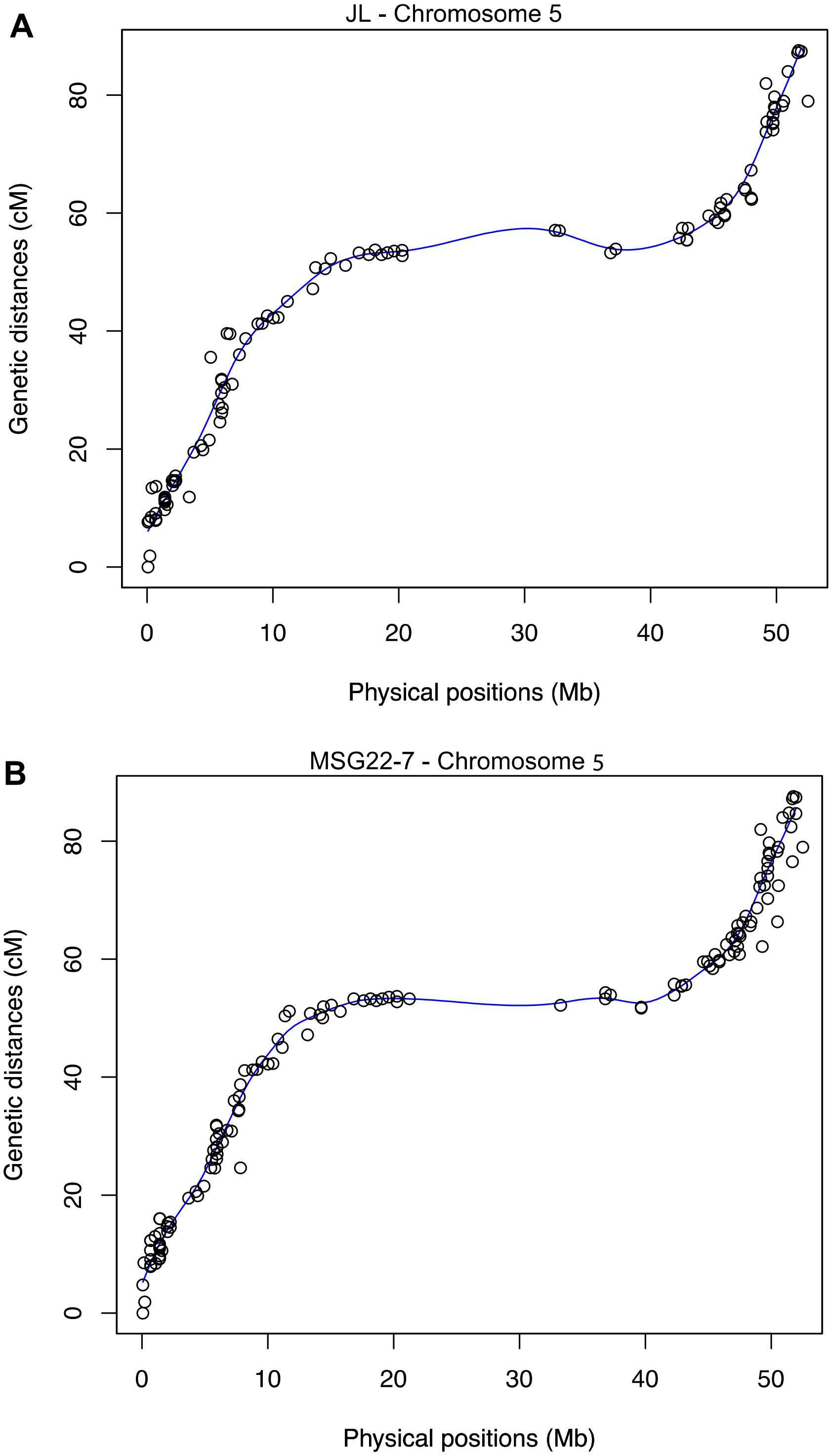

Figure S2 Graph of chromosome 5 showing the genetic location (cM) and the physical position (Mb) of SNP markers. A: "Jacqueline Lee" (JL), 104 markers. B: "MSG227-2", 140 markers.
