# Supplementary material for: Genetic Linkage Mapping of Economically Important Traits in Cultivated Tetraploid Potato (Solanum tuberosum L.)
Source: G3 (Bethesda). 2015 Sep 14;5(11):2357–64. doi: 10.1534/g3.115.019646 (PMC4632055; doi:10.1534/g3.115.019646)
Supplement: Supporting Information [file supp_g3.115.019646_TableS2.pdf]

Table S2 Parent and F1 offspring genotype configurations in the MSL603 tetraploid mapping population.

No. Clusters	Parental genotype configurations		Type	Counts
2	AAAA	AAAB	Simplex	351
	BBBB	ABBB	Simplex	352
	AAAA	ABBB	Triplex	36
	AAAB	BBBB	Triplex	28
3	AAAA	AABB	Duplex	124
	AABB	BBBB	Duplex	149
	AAAB	AAAB	Double-simplex	143
	ABBB	ABBB	Double-simplex	159
	AAAB	ABBB	X-double-simplex	59
4	AAAB	AABB	Simplex-duplex	182
	AABB	ABBB	Duplex-simplex	254
5	AABB	AABB	Double-duplex	135
**Total**				**1972**
